# Motor development following *in utero* exposure to organochlorines: a follow-up study of children aged 5–9 years in Greenland, Ukraine and Poland

**DOI:** 10.1186/s12889-015-1465-3

**Published:** 2015-02-14

**Authors:** Birgit Bjerre Høyer, Cecilia Høst Ramlau-Hansen, Henning Sloth Pedersen, Katarzyna Góralczyk, Lyubov Chumak, Bo AG Jönsson, Jens Peter Bonde, Gunnar Toft

**Affiliations:** Danish Ramazzini Centre, Department of Occupational Medicine, Aarhus University Hospital, Nørrebrogade 44, build. 2c, 8000 Aarhus C, Denmark; Department of Public Health, Section for Epidemiology, Aarhus University, Bartholins Allé 2, 8000 Aarhus C, Denmark; Primary Health Care Clinic, Postbox 570, DK-3900 Nuuk, Greenland; Department of Toxicology and Risk Assessment, National Institute of Public Health-National Institute of Hygiene, Chocimska 24, 00-791 Warsaw, Poland; Department of Social Medicine and Organization of Public Health, Kharkiv National Medical University, 61022 Kharkiv, Ukraine; Division of Occupational and Environmental Medicine, Lund University, S-221 85 Lund, Sweden; Department of Occupational and Environmental Medicine, Copenhagen University Hospital, Bispebjerg Bakke 23, 2400 Copenhagen NV, Denmark

**Keywords:** Child motor development, Developmental milestones, Dichlorodiphenyldichloroethylene (DDE), Organochlorines, Polychlorinated biphenyls (PCBs), Prenatal exposure

## Abstract

**Background:**

Prior studies on the association between prenatal exposure to polychlorinated biphenyls (PCBs) and dichlorodiphenyldichloroethylene (DDE) and child motor development have found contradicting results. Using data collected in the INUENDO cohort in Kharkiv (Ukraine), Warsaw (Poland) and Greenland (N = 1,103) between the years 2002 and 2012, we examined relations of prenatal exposure to 1,1-dichloro-2,2-bis(p-chlorophenyl)ethylene (p,p′-DDE) and 2,2′,4,4′,5,5′-hexachlorobiphenyl (CB-153) on motor development and developmental milestones; crawling, standing-up and walking.

**Methods:**

CB-153 and p,p′-DDE were measured in maternal blood in second or third trimester of pregnancy. Motor development was measured in terms of the parentally assessed screening tool Developmental Coordination Disorder Questionnaire 2007 and developmental milestones were assessed via retrospective parental reports of child age at the first time of crawling, standing-up and walking.

**Results:**

We saw no associations between tertiles of CB-153 and p,p′-DDE or log-transformed exposures and retrospective reports of the developmental milestones crawling, standing-up and walking in infancy or the motor skills measured as developmental coordination disorder at young school age.

**Conclusions:**

*In utero* exposure to CB-153 and p,p′-DDE was not associated with parentally retrospectively assessed developmental milestones in infancy or parentally assessed motor skills at young school age. The use of a more sensitive outcome measure may be warranted if subtle effects should be identified.

**Electronic supplementary material:**

The online version of this article (doi:10.1186/s12889-015-1465-3) contains supplementary material, which is available to authorized users.

## Background

Polychlorinated biphenyls (PCBs) have been widely used in industrial production, e.g. in capacitors, sealants, plasticizers, fire retardants and hydraulic fluids, but they were banned in the late 1970s in the United States and the early 1980s in Europe because of their environmental persistence [1,2]. Also, the insecticide dichlorodiphenyltrichloroethane (DDT) and its main metabolite dichlorodiphenyldichloroethylene (DDE) are highly persistent in the environment, and although the agricultural use of DDT has been severely restricted through the regional United Nations Economic Commission for Europe and the Stockholm Convention [3], it is still used as disease vector control in some developing countries [4]. PCBs and DDE are lipophilic compounds which bio-accumulate in adipose tissue and, thus, the main exposure is through food consumption and breast feeding [5,6]. The compounds pass the placental barrier and the fetus is exposed to approximately the same concentration of DDE as the mother when calculated on lipid basis [7,8]. The level of PCBs in the fetus, however, seems to be somewhat lower than that of the mother although correlated [9]. PCBs and DDE can be detected in serum and breast milk samples from almost all women around the globe, causing the fetus and subsequent breast-fed baby to be exposed to the compounds during infancy [9-12].

A major contamination of rice oil by heated PCBs and degradation products in Taiwan in 1979 gave insight into the negative impact of high prenatal exposure on e.g. neurodevelopment [13,14]. Animal studies later found decreased muscular strength [15] after high prenatal exposure to tetrachlorobiphenyl and reduced motor activity [16] after prenatal exposure to PCBs. However, results from epidemiological cohort studies are inconsistent: Higher *in utero* exposure to PCBs (3000- > 4000 μg/L) was related to a significant decrease in motor abilities indicated by 7–8 points decrease of the Psychomotor Development Index scores (PDI) in Bayley Scales of Infant Development (BSID) (a screening tool measuring children’s motor and mental abilities) compared with low exposure (0–900 μg/L) at six, 12 and 24 months in a birth cohort from North Carolina [17,18]. The Collaborative Perinatal Project from 12 hospitals in the United States found no association between background levels (0–16.50 μg/L) of prenatal PCBs exposure and motor development in terms of PDI in a sample of prenatally PCB exposed children of 8 months of age [19]. A two-point decrease in PDI scores for every 10-fold increase of DDE has been observed at six months of age, but not at 12 and 24 months [20], and further, no association was found between prenatal DDE and motor development according to McCarthy Scales of Children’s Abilities (MSCA) in two Spanish birth cohorts [21]. In addition, no association was found between *in utero* DDE exposure and motor development using BSID or MSCA among infants of one month, toddlers of 30 months or children of 3.5-5 years [22-24]. However, *in utero* exposure to DDE has been associated with hyporeflexia at one month in a cohort of North American children [25] and 4 points lower PDI for each doubling of DDE [26]. Further, no inverse associations between prenatal DDE and motor skills were observed in later follow-ups of the large North Carolina cohort [17,18].

In this cohort, we follow three populations of 1,103 children from Arctic and Europe with a large exposure span, which enables us to examine the relation between different levels of organochlorine exposures and child motor development in various parts of the world. Thus, the aim of this follow-up study was to examine the relations between prenatal exposure to p,p′-DDE and CB-153 and developmental milestones in infancy measured retrospectively and motor development assessed by the questionnaire “Developmental Coordination Disorder Questionnaire” in five to nine year old children in Greenland, Ukraine and Poland.

## Methods

### Study population

Between May 2002 and February 2004, 1,441 pregnant women were enrolled and provided a blood sample in the INUENDO (Biopersistent organochlorines in diet and human fertility) cohort from Greenland, Kharkiv (Ukraine) and Warsaw (Poland). To be eligible at baseline, the women had to be born in the country of the study, be pregnant as well as at least 18 years of age. At baseline, 2,478 women were eligible in Ukraine of whom 612 (25%) participated. In Greenland, 665 were eligible and 588 (88%) participated. All Greenlandic women were Inuits. In Poland, 690 were eligible and 258 (37%) participated. With few exceptions, the antenatal health programs covered all pregnant women in the localities.

At follow-up, 493 (81%) singleton children with measured exposure information were accessible and willing to participate in Ukraine, 525 (89%) in Greenland and 92 (36%) in Poland. A total of 1,110 children were followed-up. Further details on distribution of the study population are provided elsewhere [27].

After recording of child birth anthropometrics shortly after birth, the first follow-up of the cohort was conducted between January 2010 and May 2012 when the mean age of the children was 8 years in Greenland and Poland and 7 years in Ukraine. The retrospective reports of the developmental milestones in infancy, the children’s motor development at follow-up, and other characteristics were assessed by the parents (usually (95%) the mother), through an interview-based questionnaire. Mother-child-pairs were eligible for the study if the woman had a live-born singleton baby who was still alive at follow-up.

### Ethics statement

The study was approved by local ethical committees; Polish Bioethical Committee (approval no. 6/2002 of 3.07.2002), Ethical Committee for Human Research in Greenland (approval no. 2010–13) and the Commission on Ethics and Bioethics Kharkiv National Medical University in Ukraine (protocol number 7, October 7 2009) and all participating parents signed informed consent.

### Exposure assessment

At baseline, the women were interviewed and had a blood sample drawn. In Greenland and Kharkiv, the blood samples were drawn when the women were on average 24 weeks pregnant and in Warsaw when the women were on average 33 weeks pregnant. Ten ml cubital vein blood samples were drawn into vacuum tubes for serum collection without additives (Becton Dickinson, Maylan, France). The sera were analysed for PCBs, measured as CB-153, and DDE, measured as p,p′-DDE, and used as bio-markers of the prenatal exposure to the compounds. All chemical analyses were performed at The Department of Occupational and Environmental Medicine in Lund, Sweden. The sera were analysed by gas chromatography-mass-spectrometry following solid phase extraction. All samples were analysed twice at different days and the mean concentration of these two determinations was used. For an estimation of the imprecision in the method, the results of the analysed samples were divided into three equal sized groups, one with low levels, one with medium levels and one with the highest levels and the mean concentration for each group were calculated. Then the relative standard deviations were calculated from the duplicate determinations [28]. These were 18% at 0.1 ng/mL (n = 990), 10% at 0.5 ng/mL (n = 990) and 10% at 2 ng/mL (n = 990) for CB-153 and 11% at 1 ng/mL (n = 1058), 8% at 3 ng/mL (n = 1058) and 7% at 8 ng/mL (n = 1058) for p,p′-DDE. The detection limits were 0.05 ng/mL for CB-153 and 0.1 ng/mL for p,p′-DDE. For CB-153 there was 85 samples below the detection limit (LOD) and for p,p′-DDE there was 10 samples below LOD. If the concentration was less than LOD, the concentration was set to half the LOD based on fresh weight concentration. Sera were stored at – 20°C until analysis. CB-153 and p,p′-DDE levels were adjusted for serum concentrations of cholesterol and triglycerides which were determined by enzymatic methods. The inter-assay coefficients of variation for cholesterol and triglycerides were 1.5-2.0%. Further details are described elsewhere [29]. The laboratory was blinded concerning the measured outcomes and the mothers’ lifestyle etc. during pregnancy.

### Outcome assessment

To evaluate the motor development of the children in the early school age, we applied the country specific version of the Developmental Coordination Disorder Questionnaire 2007 (DCDQ’07). The DCDQ’07 is a parent report measure developed to assist in the identification of developmental coordination disorder in children between five and 15 years, using a five-point Likert scale. It consists of 15 items which are grouped in three factors: 1) motor control, 2) fine motor and handwriting and 3) general coordination. The sum of the age specific scores of the 15 items give an indication of whether the child suffers from a developmental coordination disorder [30]. The sum of the scores ranges from 15 to 75, lower scores indicating motor problems.

At follow-up, the parents retrospectively stated the age at which their child first crawled, stood-up with support and walked without support, using the definitions of child developmental milestones (crawling, standing-up and walking) from the Multicentre Growth Reference Study by the World Health Organization [31]. Interviewers and parents were unaware of the level of prenatal exposure of the compounds.

### Assessment of co-variates

Variables that might influence child motor development and developmental milestones and could be associated with the exposures were *a priori* identified in the literature, and data on these were harvested from questionnaires at baseline and follow-up. Baseline variables were: maternal pre-pregnancy smoking (yes/no), maternal pre-pregnancy alcohol intake (≤7, >7 servings of alcohol per week), maternal education (finished education before age 15 years, at 16–17 years, at or above 18 years), parity (1, 2–3, ≥4 child births), maternal age at the baseline interview (<30, 30–35, >35 years) and gestational age at blood drawing in weeks (continuous). Follow-up variables from immediately after birth were: sex of the child and gestational age (<37, ≥ 37 gestational week) and current follow-up variables were: breastfeeding (<6, 6–12, > 12 months) and child age at interview in years (continuous).

### Data collection

The interviews were primarily conducted face-to-face at the participants’ residence or at the local hospitals in Greenland. A medical doctor was the main interviewer in Greenland, assisted by local health workers. A telephone interview was performed when families lived in remote areas (n = 130) or participants had moved to Denmark (n = 34). In Poland, four interviewers conducted the interviews at the participant’s residence or other local meeting points. In Ukraine, all interviews were conducted at eight paediatric polyclinics by a team of 59 paediatricians. A proportion of the questionnaires were filled in by the parents without the face-to-face interview in Greenland. Also, one of the 15 items of the DCDQ was erroneously lacking in the Greenlandic version of the Questionnaire in Greenland (but not in the Danish version used for some participants in Greenland).

### Statistical analyses

#### Missing information

A relatively large amount of the parents did not fill in all items in the questionnaire, leading to missing data on outcome and co-variates. If parents left one item unanswered, it was impossible to calculate a total DCDQ score. To overcome this and take into account that complete case analysis may cause biased estimates of the association under study [32], we performed chained multiple imputations on the dataset. This approach will result in more unbiased estimates if the data are missing at random (the missingness is based on other observed characteristics such as the observed outcome or covariates) [32].

Briefly, chained multiple imputation is a statistical method that creates several new complete datasets (*m* > 1), based on known subject characteristics and other predictors in the complete dataset, incorporating the appropriate variability across the *m* datasets. The new *m* complete datasets are analysed, producing a single set of results accounting for the variability of the missing data [32].

We excluded subjects who had not filled in any of the 15 items in the DCDQ’07 from the chained multiple imputation model (n = 7), leaving a final study population of 1,103 women and children. We made 100 imputed datasets (m = 100) based on the following predictors: CB-153, p,p′-DDE, maternal education, maternal age at baseline interview, maternal pre-pregnancy smoking status, maternal pre-pregnancy alcohol consumption, parity, preterm birth, child sex, gestational age at blood drawing, child age at interview, the 15 items in the DCDQ’07, age at crawling, age at standing-up and age at walking. We performed different sensitivity analyses of the chained imputation models, including fewer and more predictors and generating less (m = 20) and more (m = 150) samples to check the robustness of the final imputation model. We present the results of the multiple imputation based analyses. Results of the complete-case analyses are available as supplementary material.

### Data analyses

Study subjects were divided into tertiles of CB-153 and p,p′-DDE exposures. CB-153 and p,p′-DDE concentrations were adjusted for serum lipids and thus expressed as ng/g lipid. As a first step in the data analysis, the correlation between CB-153 and p,p′-DDE was checked by use of Spearman’s correlation. Secondly, the crude relation between CB-153 and p,p′-DDE and motor development and developmental milestones were examined with lowest exposure tertile as the reference category. Thirdly, the adjusted relations of prenatal exposure to CB-153 and p,p′-DDE on motor development were examined by means of multiple linear regression analyses with lowest exposure tertile as the reference category. Furthermore, analyses were performed using log-transformed continuous exposures as a test for trend. The relation between *in utero* exposure to the compounds and developmental milestones was studied, using multiple linear regression analyses, with referents as above. In a sub-analysis, we excluded children born before gestational week 37. *A priori* set variables that could affect child motor development, based on literature studies, were included in the regression model and kept in the model throughout the analyses. The possible interaction between exposures and country were tested by adding an interaction term (exposures*country) to the regression models. All analyses were stratified by country as well as pooled (additionally adjusted for country).

A p-value of 0.05 or less was considered statistically significant. For all analyses the Stata statistical package was used (Version 12.1, StataCorp, College Station, Texas, USA).

## Results

A total of 1,103 of the 1,441 baseline participants were included in the follow-up analyses (77%). Although the pregnant women had to be at least 18 years of age to be included in the study, for unknown reasons, three women were 16 years old and 11 women were 17 years old in Ukraine, calculated based on the recorded date for filling in the questionnaire and date of birth.

In Greenland, Spearman’s correlation between CB-153 and p,p′-DDE was 0.9, in Poland and Ukraine, it was 0.5. The non-response analysis showed only modest differences between responders and non-responders at follow-up concerning exposure levels and no difference in maternal age at birth, birth weight and gestational age at birth (Additional file 1). Mothers were slightly older in Poland than in the other countries and in Greenland, the majority of the women were multiparae in contrast to Ukraine and Poland where most women were primiparae. Most mothers smoked prior to pregnancy in Greenland while the majority of the pregnant women from Ukraine were non-smokers. Children were youngest in Ukraine, where the mean age (SD) was 7 (1) years at follow-up. In Ukraine, median (10–90 percentile) developmental coordination disorder score (DCD) among the children <8 years of age was 47 (37–55) points and 47 (29–58) points among the children ≥8 years of age, while it was 62 (49–73) and 62 (29–58) points, respectively in Greenland and 64 (51–73) and 67 (58–74) points, respectively in Poland (Table 1).

**Table 1:** **Characteristics of mothers and their children (born 2002–2004), INUENDO cohort**

**Characteristics**	**Greenland ** **(n=520)**	**Ukraine ** **(n=492)**	**Poland ** **(n=91)**
*Mother*			
Age at baseline interview (years), mean (SD)	27 (6)	25 (5)	29 (3)
Parity, child, n (%)			
1st	164 (33)	401 (82)	86 (95)
2nd/3rd	244 (49)	91 (18)	5 (5)
4th or more	88 (18)	0 (0)	0 (0.0)
Smoking before pregnancy, n (%)			
Yes	379 (87)	112 (31)	18 (47)
No	55 (13)	250 (69)	20 (53)
Alcohol before pregnancy, n (%)			
≤7 servings/week	460 (88)	492 (100)	86 (95)
>7 servings/week	60 (12)	0 (0)	5 (5)
Educational level, Finished education at age (years), n (%)			
≤15	44 (10)	25 (6)	0 (0)
16-17	169 (36)	116 (27)	0 (0)
≥18	248 (54)	292 (67)	89 (100)
*Child*			
Sex, n (%)			
Male	280 (54)	260 (53)	54 (59)
Female	238 (46)	229 (47)	37 (41)
Age at follow-up (years), mean (SD)	8 (1)	7 (1)	8 (1)
Breastfeeding duration (months), n (%)			
0	17 (4)	42 (9)	2 (2)
<6	118 (25)	164 (33)	19 (21)
6-12	124 (26)	177 (36)	37 (41)
>12	211 (45)	107 (22)	33 (36)
Gestational age at birth (weeks), n (%)			
≥37	496 (95)	483 (98)	87 (96)
<37	24 (5)	9 (2)	4 (4)
*Exposure*			
CB-153, ng/g lipid, Median (10–90 percentile)	107 (30–369)	27 (11–54)	11 (3–24)
p,p′-DDE ng/g lipid, Median (10–90 percentile)	300 (78–959)	639 (329–1303)	440 (160–718)
*Outcome*			
Crawling (months), Mean age (SD)	6.5 (2)	6.9 (2)	9.0 (8)
Standing-up (months), Mean age (SD)	8.8 (2)	8.9 (3)	9.8 (5)
Walking (months), mean age (SD)	12.2 (2)	11.2 (1)	12.6 (4)
DCDQ-score < 8 years (points) Median [10–90 percentile]	62 [49–73]	46 [37–55]	64 [51–73]
DCDQ-score ≥ 8 years (points) Median [10–90 percentile]	62 [51–75]	47 [29–58]	67 [58–74]

*Abbreviations*: *CB-153* 2,2′,4,4′,5,5′-hexachlorobiphenyl, *DCDQ* Developmental Coordination Disorder Questionnaire, *p,p′-DDE* 1,1-dichloro-2,2-bis(*p-*chlorophenyl)-ethylene, *SD* standard deviation.

As seen in Table 2, Greenlanders were most heavily exposed to CB-153, and Ukrainians were most heavily exposed to p,p′-DDE. The median (10–90 percentile) serum levels of CB-153 not adjusted for lipids were equivalent to 0.3 (0.1-1.7) μg/L in the total population and the ditto p,p′-DDE was 3.4 (1.0-8.7) μg/L (data not shown). The proportions of missing values are presented in Table 3. Missing values range from 0 to 59%, mainly due to self-administration of a proportion of the Greenlandic questionnaires. The median missing answers in the DCDQ items were 2%.

**Table 2:** **Pregnancy tertiles of CB-153 and p,p′-DDE**

**Exposures** **ng/g lipids**	**Tertile**	**Greenland** **n = 520**	**Ukraine** **n = 492**	**Poland** **n = 91**	**All** **N = 1,103**
CB-153	Low	2.6-74.9	2.4-20.4	2.5-7.3	2.4-26.8
	Medium	75.0-167.9	20.5-34.5	7.4-13.3	26.9-75.0
	High	168.0-2,223.7	34.6-533.4	13.4-74.8	75.1-2,223.7
p,p′-DDE	Low	5.3-208.9	147.0-488.3	88.3-302.8	5.3-330.5
	Medium	209.0-445.0	488.4-790.6	302.9-471.2	330.6-636.0
	High	445.1-3,122.0	790.7-4,835.7	471.3-1,750.1	636.1-4,835.7

*Abbreviations*: *CB-153* 2,2′,4,4′,5,5′-hexachlorobiphenyl *p,p′-DDE* 1,1-dichloro-2,2-bis(*p-*chlorophenyl)-ethylene.

**Table 3:** **Number (%) of missing values according to country**

**Variables**	**Greenland (n = 520)**	**Ukraine (n = 492)**	**Poland (n = 91)**
	**n (%)**	**n (%)**	**n (%)**
*Exposures*			
Prenatal exposures	0 (0.0)	0 (0)	0 (0)
*Motor skills*			
DCDQ-score	306 (59)	11 (2)	3 (3)
Crawling	114 (22)	14 (3)	29 (32)
Standing	174 (34)	7 (1)	17 (19)
Walking	47 (9)	3 (1)	3 (3)
*Maternal and child characteristics*			
Maternal age at baseline	37 (7)	25 (5)	0 (0)
Parity	25 (5)	0 (0)	0 (0)
Smoking before pregnancy	0 (0)	0 (0)	0 (0)
Alcohol before pregnancy	0 (0)	0 (0)	0 (0)
Maternal educational level	59 (11)	59 (12)	2 (2)
Child sex	2 (0)	3 (1)	0 (0)
Breast feeding	50 (10)	3 (1)	0 (0)
Gestational age at birth	0 (0)	0 (0)	0 (0)
Child age at follow-up	52 (10)	5 (1)	0 (0)

*Abbreviations*: *DCDQ* developmental coordination disorder questionnaire.

Crude results are presented as supplementary material (Additional file 2 and Additional file 3). Medium CB-153 levels was positively associated with age at standing and walking in the total population, corresponding to approximately two weeks delay, compared to low exposed children. No associations were observed in the high exposed group, in analyses stratified by country or between DDE and either outcome.

In Table 4, results from the multiple imputation based analyses are presented, showing the adjusted mean differences of the developmental milestones crawling, standing-up and walking. We observed no associations between the exposures and the developmental milestones. Only very modest differences were seen between exposure groups in all three countries and in the pooled analysis. We found the most distinct differences of mean age at crawling in Poland, with a difference of 1.5 (95% confidence interval (CI): −5.5, 2.5) months between low and medium CB-153 exposed groups and a difference of 1.8 (CI: −5.7, 2.1) months between medium and low p,p′-DDE exposed groups. Both estimates have wide confidence intervals and are not statistically significantly different from zero, and the results comparing the high exposure group with the low exposure groups are consistent with the rest of the results, indicating no difference according to exposure levels.

**Table 4:** **Mean differences (months)** ^**a**^ **for developmental milestones in relation to maternal tertiles of CB-153 and p,p′-DDE**

		**Greenland, n = 520**			**Ukraine, n = 492**			**Poland, n = 91**			**All, n = 1,103**		
		**Diff. (95% CI)**	**Diff. (95% CI)**	**ß (95% CI)** ^**b**^	**Diff. (95% CI)**	**Diff. (95% CI)**	**ß (95% CI)** ^**b**^	**Diff. (95% CI)**	**Diff. (95% CI)**	**ß (95% CI)** ^**b**^	**Diff. (95% CI)**	**Diff. (95% CI)**	**β (95% CI)** ^**b**^
**Exposure**	**Milestone**	**Medium**	**High**	**Cont.**	**Medium**	**High**	**Cont.**	**Medium**	**High**	**Cont.**	**Medium**	**High**	**Cont.**
CB-153	Crawl	−0.1 (−0.7, 0.4)	0.0 (−0.6, 0.6)	−0.1 (−0.3, 0.2)	0.3 (−0.3, 0.8)	0.2 (−0.4, 0.8)	0.1 (−0.2, 0.5)	−1.5 (−5.5, 2.5)	−0.8 (−5.1, 3.4)	−0.1 (−2.4, 2.2)	0.2 (−0.3, 0.7)	0.1 (−0.6, 0.8)	−0.1 (−0.3, 0.2)
	Stand-up	−0.2 (−0.8, 0.4)	0.3 (−0.4, 0.9)	0.1 (−0.2, 0.3)	0.2 (−0.5, 0.9)	0.6 (−0.1, 1.3)	0.3 (−0.1, 0.7)	−0.7 (−2.7, 1.4)	1.0 (−1.4, 3.3)	0.6 (−0.7, 2.0)	0.5 (0.0, 1.0)	0.5 (−0.2, 1.1)	0.2 (−0.1, 0.4)
	Walk	−0.3 (−0.8, 0.2)	0.1 (−0.4, 0.6)	0.0 (−0.2, 0.2)	0.0 (−0.5, 0.6)	0.3 (−0.3, 0.8)	0.1 (−0.2, 0.5)	−0.1 (−1.9, 1.7)	0.6 (−1.5, 2.7)	0.7 (−0.5, 1.9)	0.4 (0.0-0.9)	0.3 (−0.2-0.9)	0.1 (−0.1, 0.3)
p,p′-DDE	Crawl	−0.1 (−0.7, 0.4)	−0.1 (−0.6, 0.5)	−0.1 (−0.3,0.1)	0.0 (−0.6, 0.5)	0.1 (−0.5, 0.7)	0.0 (−0.4, 0.4)	−1.8 (−5.7, 2.1)	−0.7 (−4.9, 3.5)	−1.1 (−3.9, 1.7)	−0.1 (−0.6, 0.4)	−0.2 (−0.8, 0.3)	−0.1 (−0.4, 0.1)
	Stand-up	−0.2 (−0.8, 0.3)	0.2 (−0.5, 0.8)	0.0 (−0.3, 0.3)	−0.2 (−0.9, 0.5)	−0.2 (−0.9, 0.6)	−0.1 (−0.6, 0.5)	0.0 (−2.1, 2.0)	0.0 (−2.3, 2.3)	0.2 (−1.4, 1.7)	0.3 (−0.2, 0.8)	0.3 2 (−0.2, 0.8)	0.0 (−0.2, 0.3)
	Walk	−0.1 (−0.6, 0.4)	−0.1 (−0.6, 0.4)	−0.1 (−0.3, 0.1)	−0.5 (−1.0, 0.1)	−0.2 (−0.7, 0.4)	−0.1 (−0.5, 0.3)	0.6 (−1.2, 2.4)	0.6 (−1.5, 2.6)	0.5 (−0.9, 1.9)	0.3 (−0.2, 0.7)	0.1 (−0.3, 0.6)	0.0 (−0.2, 0.2)

*Abbreviations*: *CB-153* 2,2′,4,4′,5,5′-hexachlorobiphenyl, *CI* confidence interval, *Cont.* Continuous, *Diff.* adjusted mean difference (months), *p,p′-DDE* 1,1-dichloro-2,2-bis(*p-*chlorophenyl)-ethylene. ^a^Adjusted for: maternal pre-pregnancy smoking, maternal pre-pregnancy alcohol-intake, maternal education, parity, maternal age at baseline interview, breast-feeding, preterm birth, gestational age at blood-sampling, child sex and child age at interview. Low exposure is the reference group. ^b^Log-transformed continuous exposure in linear regression analyses. Imputation-based analyses.

Table 5 shows results from the multiple imputation based analyses of CB-153 and p,p′-DDE and motor skills measured as developmental coordination disorder. No statistically significant differences were seen in any of the three countries or in the pooled analysis.

**Table 5:** **Mean differences (points)** ^**a**^ **for DCDQ-score in relation to pregnancy tertiles of CB-153 and p,p′-DDE**

		**Greenland, n = 520**			**Ukraine, n = 492**			**Poland, n = 91**			**All, n = 1,103**		
		**Diff. (95% CI)**	**Diff. (95% CI)**	**β** ^**b**^ **(95% CI)**	**Diff. (95% CI)**	**Diff. (95% CI)**	**β** ^**b**^ **(95% CI)**	**Diff. (95% CI)**	**Diff. (95% CI)**	**β** ^**b**^ **(95% CI)**	**Diff. (95% CI)**	**Diff. (95% CI)**	**β** ^**b**^ **(95% CI)**
**Exposure**	**Outcome**	**Medium**	**High**	**Cont.**	**Medium**	**High**	**Cont.**	**Medium**	**High**	**Cont.**	**Medium**	**High**	**Cont.**
CB-153	DCDQ	0.1 (−1.8, 2.0)	−0.6 (−2.6, 1.4)	0.2 (−0.6, 1.0)	−1.2 (−2.8, 0.4)	−0.5 (−2.2, 1.2)	−0.3 (−1.3, 0.7)	−2.7 (−8.2, 2.8)	−0.9 (−6.7, 5.0)	−0.6 (−3.9, 2.6)	0.2 (−1.1, 1.5)	0.5 (−2.2, 1.3)	−0.1 (−0.7, 0.5)
p,p′-DDE	DCDQ	−0.4 (−2.3, 1.5)	0.3 (−1.7, 2.2)	−0.1 (−0.8, 0.7)	1.1 (−0.5, 2.7)	0.6 (−1.0, 2.3)	0.6 (−0.6, 1.8)	−2.5 (−7.9, 2.9)	−2.0 (−7.8, 3.8)	−1.5 (−5.4, 2.4)	−0.5 (−1.8, 0.8)	−0.5 (−1.9, 0.9)	−0.1 (−0.7, 0.5)

*Abbreviations*: *CB-153* 2,2′,4,4′,5,5′-hexachlorobiphenyl, *CI* confidence interval, *Cont.* Continuous, *DCDQ* developmental coordination disorder questionnaire, *Diff* adjusted mean difference (points), *p,p′-DDE* 1,1-dichloro-2,2-bis(*p-*chlorophenyl)-ethylene, *Ref.* reference group. ^a^Adjusted for: maternal pre-pregnancy smoking, maternal pre-pregnancy alcohol-intake, maternal education, parity, maternal age at birth, breast-feeding, preterm birth, gestational age at blood-sampling, child sex and child age at interview. Low exposure is the reference group. ^b^Log-transformed continuous exposure in linear regression analyses. Imputation-based analyses.

When restricting our analyses to subjects with complete information, we did not observe any associations between maternal serum CB-153 and p,p′-DDE levels and either of the child’s developmental milestones in any of the three countries or in the pooled analysis (Additional file 4) except for mean age at walking that appeared to be slightly lower at higher serum p,p′-DDE levels in Greenland (p for trend, 0.04). In Poland, medium CB-153 exposed children had a lower mean age at standing compared to low exposed children; −1.0 (CI: −1.9, 0.1) months. In the complete-case analysis, results of adjusted mean differences between tertiles of CB-153 and p,p′-DDE exposures and parentally assessed motor skills at young school age are similar to the results of the multiple imputation based analyses (Additional file 5).

Using breastfeeding duration as the exposure in a sub-analysis, we found no relation to motor scores (data not shown). Excluding 37 preterm children in a sub-analysis gave roughly the same results except for a borderline statistically significant difference in Greenland. High DDE exposed children walked 0.7 months later than low exposed children (−0.7 (95% CI:-1.4; 0.0)).

The four different sensitivity analyses performed on the imputation model did not change the direction or the magnitude of the estimates (data not shown). Also, stratification by maternal education gave no clear dose–response association according to maternal education. When testing for interaction between the exposures and country, no apparent signs of interaction were found.

## Discussion

We investigated whether *in utero* exposure to p,p′-DDE and CB-153 was related to parentally retrospectively assessed delayed developmental milestones as age at crawling, standing-up and walking in infancy, and motor skills at 5–9 years of age as measured by DCDQ. We did not observe any adverse association of prenatal exposure to CB-153 and p,p′-DDE on developmental milestones or motor development.

Our results are consistent with some earlier studies with generally lower exposure levels [19,23,33]. Daniels et al. reported no association between PCBs (median (95% CI): 2.7 (1.8, 3.7) ng/ml) and motor skills in terms of BSID among 8 months old infants [19], in line with a Canadian study among 5 year-old Inuits (CB-153 mean: 99.6 ng/g lipid) [33] and a Mexican study of DDE (mean: 6.3 ng/ml) and motor skills in 30 months old children [23].

Studies with large exposure contrast between studies have found negative associations between *in utero* exposure to either or both of the compounds and parts of the motor development [17,20,25,26,34,35]. Some of the early studies on very high levels of maternal DDE (range: 0- > 6 mg/L in milk fat) and PCBs (range: 0- > 4 mg/L in milk fat) exposure and motor development in the offspring observed associations between PCBs and statistically significantly lower motor developmental scores according to BSID and between DDE and hyporeflexia according to Brazelton Neonatal Behavioral Assessment Scale in the same cohort [18,25]. The discrepancies in results could be due to the higher exposure levels and their use of other, and more detailed, outcome measure.

Among 92 toddlers, prenatal exposure to DDE has also been associated with lower PDI scores in Spain [26]. The age difference between the children of the cohorts and our use of a less detailed outcome measures could be the reason for the disparate results. A study found a 10-fold higher prenatal DDT exposure to be associated with a two-point lower psycho-motor development BSID score at six and 12 month old infants. High DDE-levels (geometric mean 1,437 ng/g lipid) were also associated to lower psycho-motor development scores at six months but not at 12 or 24 months, indicating DDT to be a psycho-motor toxic compound as well as DDE [20]. Mean DDE-levels in the study by Eskenazi et al. is more than twice the level of the highest mean level in our study and their use the more detailed BSID questionnaire, may explain the dissimilarities in results.

Breastfeeding may potentially confound the results due to its association to postnatal exposure to PCBs and DDE. On the other hand, recent studies have found breastfeeding beneficial in relation to motor development, seemingly counterbalancing the possible negative consequences of DDE [20,26]. In our data, breastfeeding does not seem to be a risk or protective factor for motor development neither in general nor when stratified by prenatal exposure levels (data not shown). We did not intend to evaluate the associations between postnatal CB-153 or DDE exposure and motor development in the present study.

In this study, the blood sample was drawn in the second or third trimester of pregnancy as did many others [19,20,34,36]. As the half-life of both compounds of interest is several years in the human body it seems unlikely that large differences in concentration appear between trimesters of pregnancy. However, high levels of first trimester DDE has been associated with motor development in infants till the age of 12 months, whereas no association was seen between second and third trimester DDE and motor development [37]. This may be a chance finding since their study, to the best of our knowledge, is the only one reporting such results. Also, Longnecker et al. found the level of DDE to be highly correlated through the trimesters of pregnancy and they concluded that the timing of measurement was not important [38].

The three diverse populations of this study have enabled us to follow children in different settings with a large exposure span. The long follow-up of up till 9 years is important since it is of importance to disclose whether possible associations between prenatal organochlorine exposure and motor skills persist into school age. In addition, these associations have rarely been investigated in these populations.

This study has some limitations. Developmental milestones were assessed retrospectively, introducing the possibility of information bias. However, we have no reason to believe that differential misclassification by exposure levels has affected the estimates as parents were unaware of their exposure levels when answering the questionnaire. However, non-differential misclassification may have attenuated the true association.

We did have a considerable proportion of missing data in the dataset, in particular on the total DCDQ-score in Greenland. In the Greenlandic version of the questionnaire (as opposed to the Danish version) one item lacked due to an error, making a calculation of the total score impossible in the Greenlandic version before imputation. Thus, we performed chained multiple imputation on the dataset because of concern for selection bias caused by missing information. Analyses on a full dataset with no missing items would have been preferable, however, when missing data does occur and data is missing at random, multiple imputation is superior to complete case analyses [39].

The median DCDQ-score was lower in Ukraine than in Greenland and Poland. This may be due to cultural differences among study sites, which has been previously reported [31]. The reliability and validity of DCDQ is acceptable [40] although previous studies have indicated that the original DCDQ may have poor sensitivity in a population sample compared to the physical motor test The Movement Assessment Battery for Children (MABC) [41] and also, the later version, DCDQ’07, had low specificity when screening an adolescent population [42]. As we intended to examine whether there was a difference in motor score according to exposure levels of DDE and CB-153, we do not believe this is of major concern for our study. In spite of a reported agreement of between 80 and 95% between DCDQ and expert opinion [40] interpretation of the prevalence of DCD in the various countries is not recommended without an additional standardized motor test to confirm the screening result of DCDQ.

Because of a relatively small study population in Poland and a substantial amount of covariates in the regression model, the results from Poland should be interpreted with caution. In Poland, the participation rates at baseline and at follow-up were rather modest (37% and 36%, respectively) and thus, the Polish part of the cohort may not fully reflect the reference population. In Ukraine, the baseline participation rate was quite low (25%) but very high at follow-up (81%). The participation rates in Greenland were very high both at baseline (88%) and at follow-up (89%) suggesting a good representation of the reference population in Greenland. Unfortunately, we were not able to compare non-participating and participating women at baseline. Comparison of respondents and non-respondents at follow-up indicate no large differences, however, loss to follow-up in particular in Poland, may have caused selection bias.

The relatively high correlation between CB-153 and DDE made us choose not to perform mutual adjustment in the analyses, and thus possible confounding of the opposite organochlorines in the results could not be eliminated.

Smoking and drinking alcohol prior to pregnancy were used as proxies for the habits during pregnancy as this information was unavailable. This may have lead to confounding of the estimates, as the women may have changed their behavior during pregnancy.

Omega-3 fatty acids may be positively related to motor development [43] and would co-occur with the exposures. Unfortunately we have no specific information on omega-3 fatty acid consumption, and thus, we cannot rule out the possibility of omega-3 fatty acid counterbalancing the possible negative effects of the organochlorines.

The results of this follow-up study show consistently no indication of an adverse effect of prenatal exposure to CB-153 and p,p′-DDE on motor development in Arctic as well as in European countries representing countries with markedly different exposure levels. However, the lack of a relation between prenatal exposures to CB-153 and DDE and motor skills may be due to generally low exposure levels or a lack of variability in range of the exposures or both, compared to earlier studies. Also, developmental retrospectively recalled milestone may not be sensible enough to detect a difference according to exposures at these levels. The use of current motor skills (DCDQ) at school age, on the other hand are not prone to recall bias.

## Conclusions

Prenatal exposure to CB-153 and p,p′-DDE were not associated with the parentally retrospectively assessed developmental milestones, age at crawling, standing and walking in infancy or parentally assessed motor skills in young school age children from Kharkiv (Ukraine), Warsaw (Poland) and Greenland, indicating no major effect of CB-153 or p,p′-DDE exposure on these outcomes. However, subtle effects cannot be excluded and the use of a more sensitive outcome measure may be warranted.

## Additional files

Additional file 1:
**Characteristics of participants and those lost to follow-up.**
Additional file 2:
**Crude mean differences (months) for developmental milestones in relation to maternal tertiles of CB-153 and p,p′-DDE.**
Additional file 3:
**Crude mean differences (points) for DCDQ-score in relation to pregnancy tertiles of CB-153 and p,p′-DDE.**
Additional file 4:
**Mean differences (months)** ^**a**^ **for developmental milestones in relation to maternal tertiles of CB-153 and p,p′-DDE.**
Additional file 5:
**Mean differences (points)** ^**a**^ **for DCDQ-score in relation to tertiles of maternal CB-153 and p,p′-DDE.**

## Abbreviations

BSID: Bayley scales of infant development
CB-153: 2,2′,4,4′,5,5′-hexachlorobiphenyl
CI: 95% confidence interval
CLEAR: Climate change, environmental contaminants and reproductive health
DCD: Developmental coordination disorder score
DCDQ′07: Developmental Coordination Disorder Questionnaire 2007
DDE: Dichlorodiphenyldichloroethylene
DDT: Dichlorodiphenyltrichloroethane
Diff: Adjusted mean difference
INUENDO: Biopersistent organochlorines in diet and human fertility
LOD: Detection limit
MABC: The movement sssessment battery for Children
MSCA: McCarthy Scales of Children’s Abilities
PDI: Psychomotor development index scores
PCBs: Polychlorinated biphenyls
p,p′-DDE: 1,1-dichloro-2,2-bis(*p*-chlorophenyl)ethylene
Ref.: Reference group
SD: Standard deviation
