# Supplementary material for: Motor development following in utero exposure to organochlorines: a follow-up study of children aged 5–9 years in Greenland, Ukraine and Poland
Source: BMC Public Health. 2015 Feb 14;15:146. doi: 10.1186/s12889-015-1465-3 (PMC4332728; doi:10.1186/s12889-015-1465-3)
Supplement: Additional file 1: — Characteristics of participants and those lost to follow-up. [file 12889_2015_1465_MOESM1_ESM.doc]

**Additional File 1.** Characteristics of participants and those lost to follow-up

|  | **Lost to follow-up**  **Greenland**  n=68 | **Participants**  **Greenland**  n=520 | **Lost to follow-up**  **Ukraine**  n=120 | **Participants**  **Ukraine**  n=492 | **Lost to follow-up**  **Poland**  n=167 | **Participants**  **Poland**  n=91 |
| --- | --- | --- | --- | --- | --- | --- |
| **CB-153, ng/g lipid,**  Median (10-90 percentile) | 148 (39-309) | 107 (30-369) | 28 (11-68) | 27 (11-54) | 11 (3-21) | 11 (3-24) |
| **p,p'-DDE ng/g lipid,**  Median (10-90 percentile) | 374 (108-663) | 300 (78-959) | 789 (344-1.649) | 639 (329-1.303) | 360 (179-785) | 440 (160-718) |
| **Maternal age years,**  mean (SD) | 28 (7) | 27 (6) | 26 (5) | 25 (5) | 29 (3) | 29 (3) |
| **Birth weight g,** mean (SD) | 3597 (591) | 3515 (996) | 3273 (419) | 3293 (524) | 3464 (474) | 3512 (531) |
| **Gestational age at birth weeks,** mean (SD) | 39.6 (1.7) | 39.2 (3.5) | 39.1 (1.2) | 39.2 (1.5) | 39.1 (1.5) | 39.3 (2.0) |

Abbreviations: CB-153; polychlorinated biphenyl congener 153, p,p´-DDE; dichlorodiphenyl dichloroetylene
