# Supplementary material for: Motor development following in utero exposure to organochlorines: a follow-up study of children aged 5–9 years in Greenland, Ukraine and Poland
Source: BMC Public Health. 2015 Feb 14;15:146. doi: 10.1186/s12889-015-1465-3 (PMC4332728; doi:10.1186/s12889-015-1465-3)
Supplement: Additional file 2: — Crude mean differences (months) for developmental milestones in relation to maternal tertiles of CB-153 and p,p′-DDE. [file 12889_2015_1465_MOESM2_ESM.doc]

**Additional file 2.** Crude mean differences (months) for developmental milestones in relation to maternal tertiles of CB-153 and p,p'-DDE

|  |  | **Greenland, n=520** | | | **Ukraine, n=492** | | | **Poland, n=91** | | | **All, n=1,103^a^** | | |
| --- | --- | --- | --- | --- | --- | --- | --- | --- | --- | --- | --- | --- | --- |
|  |  | Diff.  (95%CI) | Diff.  (95%CI) | β ^b^  (95%CI) | Diff.  (95%CI) | Diff. (95%CI) | β ^b^  (95%CI) | Diff.  (95%CI) | Diff.  (95%CI) | β ^b^  (95%CI) | Diff. (95%CI) | Diff. (95%CI) | β ^b^  (95%CI) |
| Exposure | Milestone | Medium | High | Cont. | Medium | High | Cont. | Medium | High | Cont. | Medium | High | Cont. |
| CB-153 | Crawl | -0.1  (-0.6, 0.5) | 0.1  (-0.5, 0.6) | 0.0  (-0.3, 0.2) | 0.3  (-0.3, 0.8) | 0.2  (-0.4, 0.7) | 0.1  (-0.3, 0.4) | -2.7  (-6.4, 1.1) | -1.9  (-5.6, 1.9) | -0.7  (-2.7, 1.3) | 0.2  (-0.3, 0.7) | 0.1  (-0.6, 0.8) | -0.1  (-0.3, 0.2) |
|  | Stand-up | -0.1  (-0.7, 0.4) | 0.4  (-0.2, 1.0) | 0.1  (-0.1, 0.4) | 0.1  (-0.5, 0.8) | 0.5  (-0.1, 1.2) | 0.3  (-0.2, 0.7) | -0.7  (-3.0, 1.5) | 1.1  (-1.2, 3.4) | 0.8  (-0.4, 2.0) | 0.5  (0.0, 1.0) | 0.5  (-0.1, 1.2) | 0.2  (0.0, 0.4) |
|  | Walk | -0.3  (-0.8, 0.2) | 0.1  (-0.4, 0.6) | 0.0  (-0.2, 0.3) | 0.0  (-0.6, 0.5) | 0.3  (-0.3, 0.8) | 0.1  (-0.2, 0.4) | -0.3  (-2.5, 1.8) | 0.7  (-1.5, 2.8) | 0.8  (-0.3, 1.9) | 0.4  (0.0, 0.8) | 0.3  (-0.2, 0.8) | 0.1  (-0.1, 0.3) |
| p,p’-DDE | Crawl | -0.1  (-0.6, 0.4) | 0.0  (-0.6, 0.5) | -0.1  (-0.3, 0.1) | 0.0  (-0.6, 0.5) | 0.0  (-0.5, 0.6) | -0.1  (-0.5, 0.3) | -2.2  (-5.9, 1.5) | -2.0  (-5.8, 1.8) | -1.8  (-4.3, 0.8) | -0.1  (-0.6, 0.4) | -0.3  (-0.8, 0.3) | -0.1  (-0.4, 0.1) |
|  | Stand-up | -0.2  (-0.7, 0.4) | 0.3  (-0.3, 0.9) | 0.1  (-0.2, 0.3) | -0.2  (-0.9, 0.4) | -0.2  (-0.8, 0.5) | -0.1  (-0.6, 0.4) | 0.0  (-2.3, 2.3) | 0.9  (-1.4, 3.2) | 0.8  (-0.8, 2.3) | 0.3  (-0.2, 0.8) | 0.4  (-0.2, 0.9) | 0.1  (-0.2, 0.3) |
|  | Walk | 0.0  (-0.5, 0.5) | 0.0  (-0.1, 0.4) | -0.1  (-0.2, 0.2) | -0.5  (-1.0, 0.1) | -0.2  (-0.7, 0.4) | -0.1  (-0.5, 0.3) | 0.6  (-1.5, 2.7) | 1.2  (-0.9, 3.3) | 0.9  (-0.5, 2.4) | 0.2  (-0.2, 0.6) | 0.1  (-0.3, 0.6) | 0.0  (-0.2, 0.2) |

Abbreviations: CB-153, 2,2´,4,4´,5,5´-hexachlorobiphenyl; CI, confidence interval; Cont., continuous; Diff., adjusted mean difference (months); p,p'-DDE , 1,1-dichloro-2,2-bis(*p-*chlorophenyl)-ethylene. ^a^ Adjusted for country. ^b^ CB-153 and p,p'-DDE were natural logarithm transformed in test for trend. Imputation-based analyses.
