# Supplementary material for: Motor development following in utero exposure to organochlorines: a follow-up study of children aged 5–9 years in Greenland, Ukraine and Poland
Source: BMC Public Health. 2015 Feb 14;15:146. doi: 10.1186/s12889-015-1465-3 (PMC4332728; doi:10.1186/s12889-015-1465-3)
Supplement: Additional file 3: — Crude mean differences (points) for DCDQ-score in relation to pregnancy tertiles of CB-153 and p,p′-DDE. [file 12889_2015_1465_MOESM3_ESM.doc]

**Additional file 3.** Crude mean differences (points) for DCDQ-score in relation to pregnancy tertiles of CB-153 and p,p'-DDE

|  |  | **Greenland, n=520** | | | **Ukraine, n=492** | | | **Poland, n=91** | | | **All, n=1,103^a^** | | |
| --- | --- | --- | --- | --- | --- | --- | --- | --- | --- | --- | --- | --- | --- |
|  |  | Diff.  (95%CI) | Diff.  (95%CI) | β ^b^  (95%CI) | Diff.  (95%CI) | Diff. (95%CI) | β ^b^  (95%CI) | Diff.  (95%CI) | Diff.  (95%CI) | β ^b^  (95%CI) | Diff. (95%CI) | Diff. (95%CI) | β ^b^  (95%CI) |
| Exposure | Outcome | Medium | High | Cont. | Medium | High | Cont. | Medium | High | Cont. | Medium | High | Cont. |
| CB-153 | DCDQ | -0.2  (-2.1, 1.6) | -0.8  (-2.7, 1.1) | 0.0  (-0.7, 0.8) | -1.0  (-2.6, 0.6) | -0.6  (-2.2, 1.0) | -0.4  (-1.4, 0.6) | -1.2  (-6.7, 4.4) | -1.1  (-6.7, 4.4) | -1.1  (-3.9, 1.8) | 0.1  (-1.2, 1.4) | -0.7  (-2.4, 1.0) | -0.2  (-0.7, 0.4) |
| p,p’-DDE | DCDQ | -0.4  (-2.3, 1.4) | 0.0  (-1.9, 1.8) | -0.2  (-0.9, 0.5) | 1.3  (-0.3, 2.9) | 0.8  (-0.8, 2.4) | 0.6  (-0.6, 1.8) | -0.7  (-6.2, 4.8) | -1.6  (-7.1, 3.9) | -1.8  (-5.6, 1.9) | -0.3  (-1.6, 1.0) | -0.5  (-1.8, 0.9) | -0.1  (-0.7, 0.5) |

Abbreviations: CB-153, 2,2´,4,4´,5,5´-hexachlorobiphenyl; CI, confidence interval; DCDQ, developmental coordination disorder questionnaire; Diff, adjusted mean difference (points); p,p'-DDE , 1,1-dichloro-2,2-bis(*p-*chlorophenyl)-ethylene; Ref., reference group. ^a^ Adjusted for country. Low exposure is the reference group. ^b^ CB-153 and p,p'-DDE were natural logarithm transformed in test for trend. Imputation-based analyses.
