# Supplementary material for: Motor development following in utero exposure to organochlorines: a follow-up study of children aged 5–9 years in Greenland, Ukraine and Poland
Source: BMC Public Health. 2015 Feb 14;15:146. doi: 10.1186/s12889-015-1465-3 (PMC4332728; doi:10.1186/s12889-015-1465-3)
Supplement: Additional file 4: — Mean differences (months) a for developmental milestones in relation to maternal tertiles of CB-153 and p,p′-DDE. [file 12889_2015_1465_MOESM4_ESM.doc]

**Additional file 4.** Mean differences (months)^a^  for developmental milestones in relation to maternal tertiles of CB-153 and p,p'-DDE

|  |  | **Greenland** | | | | **Ukraine** | | | |
| --- | --- | --- | --- | --- | --- | --- | --- | --- | --- |
|  |  |  | Diff. (95%CI) | Diff. (95%CI) | β (95%CI) ^b^ |  | Diff. (95%CI) | Diff. (95%CI) | β (95%CI) ^b^ |
| Exposure | Milestone | n | Medium^c^ | High^c^ | Cont. | n | Medium^c^ | High^c^ | Cont. |
| CB-153 | Crawl | 275 | 0.1 (-0.4, 0.6) | 0.2 (-0.3, 0.8) | 0.0 (-0.2, 0.2) | 366 | 0.0 (-0.3, 0.3) | 0.2 (-0.1, 0.5) | 0.1 (-0.1, 0.3) |
|  | Stand-up | 223 | 0.1 (-0.7, 0.9) | 0.5 (-0.3, 1.3) | 0.1 (-0.3, 0.4) | 371 | 0.1 (-0.2, 0.4) | 0.3 (0.0, 0.6) | 0.2 (0.0, 0.4) |
|  | Walk | 317 | -0.5 (-1.1, 0.1) | -0.3 (-0.9, 0.4) | -0.2 (-0.4, 0.1) | 375 | 0.0 (-0.4, 0.4) | 0.1 (-0.3, 0.4) | 0.0 (-0.2, 0.3) |
| p,p’-DDE | Crawl | 273 | 0.0 (-0.5, 0.4) | 0.0 (-0.4, 0.5) | -0.1 (-0.2, 0.1) | 366 | 0.1 (-0.2, 0.4) | -0.2 (-0.5, 0.2) | -0.1 (-0.3, 0.1) |
|  | Stand-up | 223 | 0.2 (-0.6, 1.0) | 0.4 (-0.5, 1.2) | 0.0 (-0.3, 0.4) | 371 | 0.2 (-0.1, 0.5) | 0.2 (-0.1, 0.5) | 0.1 (-0.1, 0.3) |
|  | Walk | 299 | -0.2 (-0.9, 0.4) | -0.6 (-1.3, 0.1) | -0.3 (-0.5, 0.0) | 374 | -0.2 (-0.5, 0.2) | 0.0 (-0.4, 0.4) | -0.1 (-0.3, 0.2) |
|  |  | | **Poland** |  |  |  | **All** |  |  |
|  |  | | Medium | High |  |  | Medium | High |  |
| CB-153 | Crawl | 59 | -0.5 (-2.1, 1.0) | 0.5 (-1.1, 2.2) | 0.6 (-0.4, 1.7) | 698 | 0.1 (-0.2, 0.4) | 0.3 (-0.2, 0.7) | 0.0 (-0.1, 0.2) |
|  | Stand-up | 68 | -1.0 (-1.9, 0.1) | 0.1 (-0.9, 1.1) | 0.0 (-0.7, 0.7) | 658 | 0.1 (-0.2, 0.4) | 0.4 (-0.2, 0.9) | 0.1 (-0.1, 0.3) |
|  | Walk | 81 | 0.0 (-0.9, 1.0) | 0.0 (-1.0, 1.0) | 0.1 (-0.5, 0.7) | 765 | 0.2 (-0.2, 0.5) | -0.1 (-0.6, 0.4) | -0.1 (-0.2, 0.1) |
| p,p’-DDE | Crawl | 56 | -1.0 (-2.2, 0.3) | -1.0 (-2.2, 0.6) | 0.0 (0.0, 0.0) | 690 | 0.1 (-0.2, 0.3) | -0.2 (-0.5, 0.1) | -0.1 (-0.2, 0.0) |
|  | Stand-up | 68 | 0.3 (-0.6, 1.3) | -0.4 (-1.5, 0.6) | -0.3 (-1.0, 0.5) | 658 | 0.1 (-0.3, 0.5) | 0.0 (-0.4, 0.4) | 0.1 (-0.1, 0.2) |
|  | Walk | 80 | 0.3 (-0.7, 1.3) | -0.3 (-1.4, 0.7) | -0.2 (-1.0, 0.6) | 745 | 0.0 (-0.4, 0.4) | -0.1 (-0.5, 0.3) | -0.1 (-0.3, 0.0) |

Abbreviations: CB-153, 2,2´,4,4´,5,5´-hexachlorobiphenyl; CI, confidence interval; Cont., continuous; Diff, adjusted mean difference (months); p,p'-DDE, 1,1-dichloro-2,2-bis(*p-*chlorophenyl)-ethylene. ^a^ Adjusted for: maternal pre-pregnancy smoking, maternal pre-pregnancy alcohol-intake, maternal education, parity, maternal age at baseline interview, breast-feeding, preterm birth, gestational age at blood-sampling, child sex and child age at interview. ^b^ CB-153 and p,p'-DDE were natural logarithm transformed in test for trend. ^c^ Low exposure is reference group. Complete case analyses.
