# Supplementary material for: Motor development following in utero exposure to organochlorines: a follow-up study of children aged 5–9 years in Greenland, Ukraine and Poland
Source: BMC Public Health. 2015 Feb 14;15:146. doi: 10.1186/s12889-015-1465-3 (PMC4332728; doi:10.1186/s12889-015-1465-3)
Supplement: Additional file 5: — Mean differences (points) a for DCDQ-score in relation to tertiles of maternal CB-153 and p,p′-DDE. [file 12889_2015_1465_MOESM5_ESM.doc]

**Additional file 5.** Mean differences (points)^a^  for DCDQ-score in relation to tertiles of maternal CB-153 and p,p’-DDE

|  | **Greenland** | | | | **Ukraine** | | | |  |
| --- | --- | --- | --- | --- | --- | --- | --- | --- | --- |
|  |  | Diff. (95%CI) | Diff. (95%CI) | β (95%CI)^b^ |  | Diff. (95%CI) | Diff. (95%CI) | β (95%CI) ^b^ | |
| Exposure | n | Medium^c^ | High^c^ | Cont. | n | Medium^c^ | High^c^ | Cont. | |
| CB-153 | 152 | 1.2 (-2.1, 4.5) | 0.1 (-3.6, 3.7) | 0.8 (-0.8, 2.3) | 369 | -1.4 (-3.2, 0.5) | -0.3 (-2.1, 1.5) | 0.2 (-1.0, 1.4) | |
| p,p’-DDE | 152 | 0.5 (-0.3, 3.9) | 0.1 (-3.5, 3.6) | 0.4 (-0.9, 1.8) | 369 | 1.4 (-0.3, 3.2) | 1.6 (-0.3, 3.4) | 0.6 (-0.8, 2.0) | |
|  |  | **Poland** | |  |  | **All** | |  | |
| CB-153 | 81 | -3.2 (-9.5, 3.1) | -3.1 (-9.6, 3.4) | -1.7 (-6.1, 2.8) | 594 | -0.7 (-2.3, 0.9) | -0.3 (-3.1, 2.5) | 0.1 (-0.8, 1.0) | |
| p,p’-DDE | 81 | -2.4 (-8.5, 3.6) | -1.9 (-7.9, 4.2) | -1.0 (-6.6, 4.5) | 594 | -0.5 (-2.3, 1.4) | 0.1 (-1.9-2.1) | 0.2 (-0.7, 1.2) | |

Abbreviations: CB-153, 2,2´,4,4´,5,5´-hexachlorobiphenyl; CI, confidence interval; Cont., continuous; DCDQ, developmental coordination disorder questionnaire; Diff., adjusted mean difference (points); p,p'-DDE;, 1,1-dichloro-2,2-bis(*p-*chlorophenyl)-ethylene. ^a^ Adjusted for: maternal pre-pregnancy smoking, maternal pre-pregnancy alcohol-intake, maternal education, parity, maternal age at birth, breast-feeding, preterm birth, gestational age at blood-sampling, child sex and child age at interview. Low exposure is reference group. ^b^ CB-153 and p,p'-DDE were natural logarithm transformed in test for trend. ^c^ Low exposure is reference group. Complete-case analyses.
